# Diet in Disease

**Published:** 1893-03-11

**Authors:** Ernest Hart

**Affiliations:** Bachelier-ès-Sciences-es-Lettres (rèstreint), formerly Student of the Faculty of Medicine of Paris, and of the London School of Medicine for Women


### Diet in Disease.

By Mrs. Ernest Hart, Bachelier-ès-Sciences-es-Lettres (rèstreint), formerly Student of the Faculty of Medicine of Paris, and of the London School of Medicine for Women.

### XV.—DIABETES (*continued*).

### A WEEK'S *MENUS* FOR A DIABETIC.

(*Time—Winter.*)

First Day.

*Breakfast.*

Smoked salmon.
Kidneys and bacon, on a bed of Brussels sprouts.
Nib cocoa and cream.

*Lunch.*

Roast grouse.
Baked custard pudding, with bottled green fruit stewed with saccharine. (1)
Brie cheese.

*Dinner.*

Celery soup.
Red mullet en papillottes.
Spanish steaks. (2)
Tomatoes, with frozen savoury cream. (3)
Russian caviare on gluten croutons.

*Recipes.*

(1) Bottled Green Fruit.—The fruit—greengages, plums, gooseberries, cherries, currants, &c., should be gathered when green and unripe, bottled in the usual way, and sweetened with saccharin.

(2) Spanish Steaks.—Take one pound of rump steak, two onions, two ounces of butter, one and a-half gill of brown stock hot, half a tablespoonful of tarragon vinegar, and one tablespoonful of cream. Cut the steak into rounds, score them with a knife, sprinkle parsley on the top of each, then put a little butter and vinegar over them; let them stand while the sliced onion is frying in the butter. Strain the steaks, see that no fat is left in stewpan, cover the bottom of it with the hot stock, let the steaks simmer gently in the stock for one hour, then dish them. Reduce the gravy by stewing to half the quantity, pour it round the steaks, adding the onion before serving.

(3) Cut fresh tomatoes in half, place them on ice till slightly frozen, whip a proportionate amount of cream, mix with it pepper, salt, and a little tarragon vinegar, ice, and place a dessert spoonful on the cut surface of each tomato.

Second Day.

*Breakfast.*

Herrings and mustard sauce.
Cold tongue.
Callard's almond biscuits.
Coffee and cream.

*Lunch.*

Oyster omelette.
Roast loin of mutton with mashed turnips.
Gorgonzola cheese.

*Dinner.*

Clear soup and grated Parmesan cheese.
Steamed turbot with Dutch sauce.
Braised pheasant with purée of savoy cabbage.
Seakale with French butter.

Third Day.

*Breakfast.*

Cod roes, stewed brown. (4)
Swiss eggs. (5)
Potash water and cream.

*Lunch.*

Roast rabbit with stewed leeks.
Rhubarb and cream.
Stilton cheese and Callard's cheese biscuits.

*Dinner.*

Oysters.
Soup, Croûte au pot. (6)
Roast goose and broccoli.
Stewed celery.
Cocoanut cream. (7)

*Recipes.*

(4) Fresh Cod Roes are rather neglected; they can be purchased for a few pence, and are excellent for breakfast stewed brown. Parboil them first; let them get cold; cut in slices, and stew in a rich brown gravy. They make also a light pleasant dish, fried in cutlets.

(5) Swiss Eggs.—Spread 2 oz. of butter on the bottom of a fire-proof porcelain dish, and lay on it six thin slices of Gruyère cheese; break six eggs on this, keeping the yolks whole. Sprinkle over some mignonette pepper and salt. Mix together a tablespoonful of chopped parsley and 2 oz. of grated Gruyere cheese. Strew over the eggs. Bake in a quick oven from ten to twelve minutes. Serve in the dish they are baked in.

(6) Croute au Pot.—The croutons must be made of gluten bread.

(7) Cocoanut Cream.—Whip cream and mix fresh grated cocoanut with it; sweeten with saccharin if required.

Fourth Day.

*Breakfast.*

Fried bacon, served on a purée of Brussels sprouts.
Cold pheasant.
Van Houten's cocoa and cream.

*Lunch.*

Braised leg of Welsh mutton, with tomatoes and mushrooms. (8)
Cheese cake (9) and cream cheese.

*Dinner.*

Hare soup, without wine.
Water souché of sole.
Duck, with turnips. (10)
Russian salad (leaving out the potatoes, carrots, and peas).

*Recipes.*

(8) The mutton is braised as already described. The tomatoes and mushrooms are cooked in the oven with a little butter, and placed round the dish.

(9) Cheese Cakes.—One pint of milk, half a tablespoonful of rennet, one ounce of butter, two eggs, one tablespoonful of brandy, quarter of an ounce of almonds, and saccharin. Turn the milk to a curd; let it stand in a warm place till thoroughly set, tie a piece of muslin over a bowl, break up the curd and pour it on to the muslin; leave it till all the whey has run off. Beat the curd smooth and add the butter and eggs well beaten with the brandy, almonds, and saccharin. When well mixed pour some of the mixture into each of the patty pans and bake for about 15 to 20 minutes.

(10) Duck with Turnips.—Slightly brown the duck in a flat stew pan with a little butter and onion; then add a pint of good stock well flavoured with vegetables, herbs, spices, &c. Keep the stew pan well closed so that the steam does not escape. Simmer gently for 1½ to 2 hours. Remove the fat from the gravy and serve with the duck. The turnips are cut in thin slices, fried gently in butter, and served in the dish with the duck.

Fifth Day.

*Breakfast.*

Ham or tongue omelette.
Spiced beef.
Tea and cream.

*Lunch.*

Larded sweetbread.
Cold mutton with endive salad.
Camembert cheese.

*Dinner.*

Lettuce soup. (11)
Fried cutlets of cod.
Sirloin of beef with gherkins (12) and stewed Scotch kale.
Devonshire junket. (13)

*Recipes.*

(11) Lettuce Soup.—This is made the same way as spinach soup, only winter lettuce is used.

(12) Sirloin of Beef with Gherkins.—Roast the beef; when half cooked add the vinegar from a bottle of gherkins to the dripping, and baste constantly. Chop the gherkins quite small, and place them round the dish in the gravy when served.

(13) Make the junket in the usual way; add a solution of saccharin to the whipped cream. The saccharin must not be added to the milk, else the rennet will not make curds.

Sixth Day.

*Breakfast.*

Stewed mushrooms.
Cold fowl.
Boiled eggs.

*Lunch.*
Haricot oxtail.
Coffee cream.

*Dinner.*
Oyster stew.
Marinaded venison cutlets.
Boiled guinea-fowl, with celery sauce.
Little tarragon creams (14).

(14) Put into a basin one white and two yolks of eggs, a quarter of a pint of cream, a little white pepper and salt. Beat up well with a fork till smooth, and add a little chopped tarragon. Butter some little dariole moulds and sprinkle them with chopped tarragon and truffles mixed. Pour in the cream mixture and stand the darioles in a stewpan of boiling water reaching to three-quarters of the height of the moulds. When the water boils draw the pan to the side of the stove and poach for about twenty to thirty minutes till the creams are set. Turn out on to a warm dish, and serve with cream sauce round them. The cream sauce is prepared by putting into a stewpan one oz. of butter, two raw yolks of eggs, four tablespoonfuls of thin cream, a pinch of salt, and three or four drops of lemon juice. Stir in a bain-marie till the sauce thickens, add a saltspoonful of tarragon vinegar, and strain it. Mix in a light sprinkling of fresh tarragon and serve. This cream sauce will be found nice to serve with fillets of soles, whiting, &c.

SEVENTH DAY.

*Breakfast.*
Finnan haddock.
Cold brawn.
Cocoatina and cream.

*Lunch.*
Boiled calf's head.
Turnip tops.

*Dinner.*
Clear soup with poached eggs.
Fried cutlets of plaice.
Roast turkey. Broccoli sprouts.
Fish-roe soufflés. (15)

*Recipe.*

(15) FISH ROE SOUFFLÉS.—Take six soft roes of fresh herrings; blanch, pound, and tammy them; then flavour with salt, pepper, powdered mace and nutmeg; add half-an-ounce of butter and the yolks of two eggs; beat well together; whisk the whites of six eggs into a stiff froth, mix same with the roes, and bake in rammakin cups for about five minutes. Serve immediately the soufflés are removed from oven.

